# Is rapid antibacterial susceptibility testing medium reliable for routine laboratory practices?

**DOI:** 10.12669/pjms.312.6683

**Published:** 2015

**Authors:** Gulhan Yagmur, Baris Derya Ercal, Zafer Mengeloglu, Fatma Mutlu Sariguzel, Elife Berk, Derya Saglam

**Affiliations:** 1 Gulhan Yagmur, Department of Postmortem Microbiology, Council of Forensic Medicine, Istanbul, Turkey; 2 Baris Derya Ercal, Department of Medical Microbiology, Erciyes University Medical Faculty, Kayseri, Turkey; 3 Zafer Mengeloglu, Abant Izzet Baysal University Medical Faculty, Department of Medical Microbiology, Bolu, Turkey; 4 Fatma Mutlu Sariguzel, Department of Microbiology, Kayseri Training and Research Hospital, Kayseri, Turkey; 5 Elife Berk, Department of Medical Microbiology, Erciyes University Medical Faculty, Kayseri, Turkey; 6 Derya Saglam, Department of Microbiology, Kayseri Training and Research Hospital, Kayseri, Turkey

**Keywords:** Antibacterial susceptibility, Disk diffusion, Quicolor agar

## Abstract

**Objective:**

Early detection of antibiotic susceptibility profile of the isolates has critical importance in terms of immediate beginning of the appropriate treatment and increasing of treatment success, such as meningitis, bacteriemia and sepsis. In the present study, it was aimed to compare the antibiotic susceptibility results of Quicolor (Salubris Inc., Massachusetts, USA) and standard disk diffusion method.

**Methods:**

One hundred twenty three isolates were included in this study (80 Enterobacteriaceae, 15 Staphylococci and 28 nonfermentative Gram-negative bacteria). Antibiotic susceptibility in clinical isolates was evaluated using Mueller-Hinton (MH) agar and Quicolor (ES and NF) agar plates.

**Results:**

For Enterobacteriaceae, frequency of total concordance, major error, and minor error between the tests were found as 96.8%, 0.8%, and 2.4%, respectively. For Staphylococci, frequency of total concordance, major error, and minor error among the tests were found as 95.7%, 3.5%, and 0.8%, respectively. For non fermentative bacteria, frequency of total concordance, major error, and minor error among the tests were found as 83.9%, 9.6%, and 6.4%, respectively.

**Conclusions:**

Quicolor media provided reliable susceptibility results in enteric bacteria and Staphylococci. However, further studies including higher number of nonfermentative bacteria are required to determine whether the chromogenic media are appropriate for this group of bacteria.

## INTRODUCTION

Antibiotic resistance in microorganisms is still an important health problem worldwide. Becoming resistant and capability of transferring resistance factors to other bacteria make it difficult to fight off diseases and gradually reduce the treatment options.^1^,^2^

Early detection of antibiotic susceptibility profile of the isolates obtained from patients, particularly from intensive care units, who have life-threating manifestations such as meningitidis, bacteriemia and sepsis as well as more common and less severe infections has critical importance in terms of immediate beginning of the appropriate treatment and increasing of treatment success. In such cases, the success of the treatment would reduce the side effects of antibiotic, mortality and treatment costs.^3^-^6^

In routine microbiology laboratory, antibiotic susceptibility testing is performed using either disk diffusion method or automated systems, and it should be expected for the results for approximately 16-24 hours. Some molecular methods are available for determination of the susceptibility profile in a shorter period. However, using these methods in routine laboratory are not cost-effective, therefore, researchers should work on some low-cost methods that are also easier to perform. It is reported that expecting time has been reduced to 4-6 hours with some recent methods in which chromogenic media are used.^7^-^9^

In the present study, we aimed to compare the antibiotic susceptibility results of Quicolor (Salubris Inc., Massachusetts, USA) and standard disk diffusion method, and to investigate reliability of this chromogenic medium.

## METHODS

**Fig.1:**
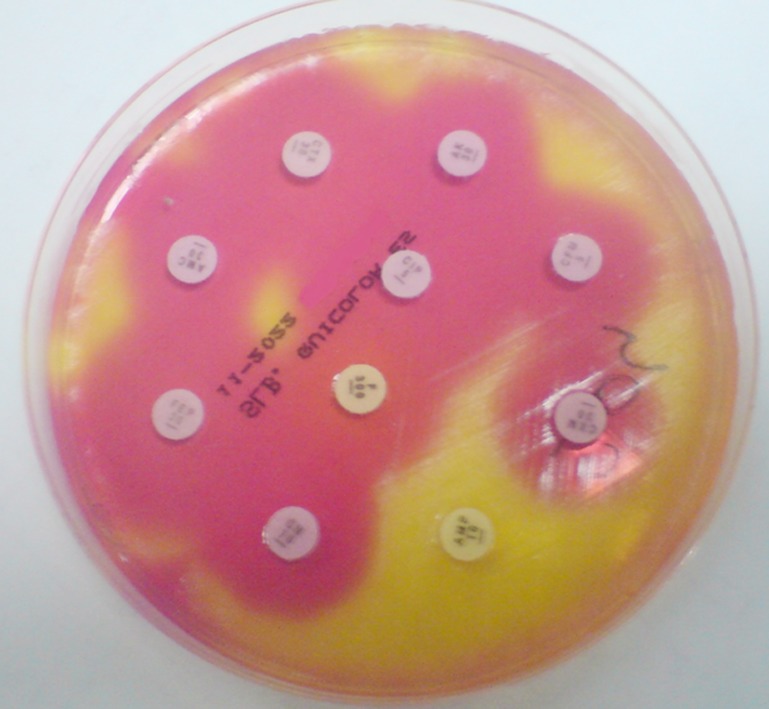
A sample for Quicolor media.

This study was approved by the local ethics committee. A total of 123 isolates consisting of 80 Enterobacteriaceae isolates (70 *Escherichia coli*, 5 *Klebsiella pneumoniae*, 1 *Klebsiella oxytoca*, 2 *Proteus spp*., 1 *Morganella morganii*, 1 *Serratia marcescens*), 15 Staphylococci (7 *Staphylococcus aureus*, 7 *Staphylococcus epidermidis*, 1 *Staphylococcus saprophyticus*) and 28 nonfermentative Gram negative bacteria (20 *Pseudomonas aeruginosa*, 8 *Acinetobacter spp*.) all of which were obtained from various clinical specimens in the microbiology laboratory of Kayseri Training and Research Hospital were included in the study. Bacterial suspensions were adjusted to a density of 0.5 McFarland standard, and were inoculated onto both Mueller Hinton (MH) media (bioMérieux, France) and Quicolor (Salubris Inc., Massachusetts, USA) chromogenic media, and antibiotic disks were placed onto the inoculated media. All media were incubated at 35°C, MH media for 18-24 hours, and Quicolor media for approximately 6-10 hours until the color changes at the inhibition zone became visible. At the end of the incubation period, inhibition zones around the disks on the Salubris media were measured and evaluated according to the manufacturer’s recommendations (Fig.1), and the zone diameters on the MH media were evaluated according to the criteria of the Clinical and Laboratory Standards Institute (CLSI 2014)

The evaluation of the results was completed at the end of 6th hour of incubation for enteric bacteria and staphylococci, and it was done at 9th or 10th hours of incubation for nonfermentative ones. If the test results were the same, either susceptible or resistant by both tests, it was defined as “concordance”. If the result was susceptible or resistant by one test and intermediate with the other, it was defined as “minor error”. If the result was susceptible by one test and resistant by the other it was defined as “major error”. *E. coli* ATCC 25922, *S. aureus* ATCC 25923, *P. aeruginosa* ATCC 27853 were evaluated as quality control strains.

## RESULTS

For Enterobacteriaceae, frequency of total concordance, major error, and minor error between the tests were found as 96.8%, 0.8%, and 2.4%, respectively. For staphylococci, frequency of total concordance, major error, and minor error between the tests were found as 95.7%, 3.5%, and 0.8%, respectively. For nonfermentative bacteria, frequency of total concordance, major error, and minor error between the tests were found as 83.9%, 9.6%, and 6.4%, respectively. In the enteric bacteria group, the highest minor error frequency was found in eight isolates (10%) for amoxicillin/clavulanate, the highest major error rate was found in three isolates (3.7%) for gentamicin. In nonfermentative group, the highest minor error frequency was found in five isolates (17.8%) for cefoperazone/sulbactam, the highest major error was observed in four isolates (14.2%) for amikacin (Table-I, II, and III).

**Table-I:** Concordance rates between Quicolor and standard disk diffusion methods for Enterobacteriaceae (n=80).

Antibiotics	Minor error n (%)	Major error n (%)	Total error n (%)	Concordance n (%)
Ampicillin	3 (3.7%)		3 (3.7%)	77 (96.3%)
Amoxicillin/clavulanate	8 (10.0%)	1 (1.2%)	9 (11.2%)	71 (88.8%)
Cefuroxim axetil	1 (1.2%)		1 (1.2%)	79 (98.2%)
Cefepim	2 (2.5%)	1 (1.2%)	3 (3.7%)	77 (96.3%)
Gentamicin	1(1.2%)	3 (3.7%)	4 (5.0%)	76 (95.0%)
Amikasin				80 (100%)
Ciprofloxasin				80 (100%)
Meropenem				80 (100%)
Total	15 (2.4%)	5 (0. 8%)	20 (3.2%)	620 (96.8%)

**Table-II:** Concordance rates between Quicolor and standard disk diffusion methods for staphylococci (n=15).

Antibiotics	Minor error n (%)	Major error n (%)	Total error n (%)	Concordance n (%)
Cefoxitin		1(6.6%)	1(6.6%)	14 (94.3%)
Eritromycin				15 (100%)
Clindamycin				15 (100%)
Ciprofloxasin				15 (100%)
Gentamicin		1(6.6%)	1(6.6%)	14 (94.3%)
Amikacin	1(6.6%)		1(6.6%)	14 (94.3%)
Vancomycin		1(6.6%)	1(6.6%)	14 (94.3%)
Teicoplanin		1(6.6%)	1(6.6%)	14 (94.3%)
Total	1 (0.8%)	4 (3.5%)	5 (4.3%)	115 (95.7%)

**Table-III:** Concordance rates between Quicolor and standard disk diffusion methods for nonfermentative bacteria (n=28).

Antibiotics	Minor error n (%)	Major error n (%)	Total error n (%)	Concordance n (%)
Cefoperazone/sulbactam	5 (17.8%)	2 (7.1%)	7 (24.9%)	21 (75.1%)
Imipenem	2 (7.1%)	2 (7.1%)	4 (14.2%)	24 (86.8%)
Ceftazidim	2 (7.1%)	3 (10.7%)	5 (17.8%)	23 (82.2%)
Cefepim	2 (7.1%)	3 (10.7%)	5 (17.8%)	23 (82.2%)
Gentamicin		3 (10.7%)	3 (10.7%)	25 (89.3%)
Netilmycin				28 (100%)
Amikacin		4 (14.2%)	4 (14.2%)	24 (86.8%)
Ciprofloxacin		3 (10.7%)	3 (10.7%)	25 (89.3%)
Meropenem	3 (10.7%)	1 (3.5%)	4 (14.2%)	24 (86.8%)
Total	14 (6.4%)	21 (9.6%)	35 (16.1%)	217(83.9%)

## DISCUSSION

Recently, rapid antimicrobial susceptibility methods have been used with a capability of providing results in 4-12 hours in microbiology laboratories. These methods have higher cost than standard disk diffusion method, but they are cheaper than molecular tests.^10^,^11^ Chromogenic Quicolor disk diffusion medium is considered as an inexpensive method that can be preferred to be used for rapid susceptibility results particularly in critical patients with meningitis, bacteremia, or sepsis.^7^,^12^

In the present study, it was observed that Quicolor test results were provided in a period shorter than 10 hours, and less than six hours for enteric bacteria and staphylococci. These periods mean that the susceptibility results can be provided at the same day, and it can be considered to contribute early beginning of the appropriate treatment. However, some discrepancies were found between the results of the two methods. The total concordance of 95% shows that the chromogenic media can be reliable for enteric bacteria and staphylococci. However, total concordance was found about 80% for nonfermentative bacteria that include *P. aeruginosa* and *A. baumannii* both of which are very common causative agents of nosocomial infections particularly in intensive care units. Besides this, for nonfermentatives, major error rate was found about 10%, and this makes us consider that chromogenic media are not reliable enough for this group of bacteria.

Ercis et al.^13^ investigated the reliability of Quicolor media in detection of extended-spectrum beta-lactamase (ESBL) production of bacteria using disks and strips containing various antimicrobial combinations with disk diffusion and Etest methods, and they reported that the results provided at the end of a 4-6 hours of incubation showed a total concordance of 97%.

Kocagöz et al.^14^ compared presence of ESBL in 184 *Salmonella* isolates using double-disk synergy method with Quicolor and MH media, and they reported positivity in six isolates and that the both methods showed complete concordance with each other.

Kocagöz et al.^15^ reported the total concordance as 97.6% in all isolates, 96.7% in enteric bacteria, 96.8% in staphylococci, and 94.2% in norfermentative bacteria in their study conducted for comparison of reliability of Quicolor media with a total of 177 isolates. They also reported very low major error rates between 0.6-1.7%. The concordance rates of Kocagöz et al.^14^ are very close to our data in enteric bacteria and staphylococci groups. However, our concordance rate was lower, and our major error rate was higher than their rates in nonfermentative group. These discrepancies can be due to our lower number of isolates. Moreover, due to lack of molecular methods determining the clonal relationship amongst the isolates in both studies, epidemic clones might be included in the tests, and this condition, particularly the number of probable epidemic clones, might have affected the concordance frequency between the studies. For instance, in a study including a multi-resistant clone consisting of most of the isolates, the resistance rate would be found higher as it was, and this clone would found as resistant using all the methods, and concordance rate between methods would be calculated in a high rate. Including high number of isolates in the studies lack of determination of clonal relatedness can reduce this probability in very low rates.

Coban et al.^16^ aimed to determine susceptibility break points for methicillin resistance in *Staphylococcus aureus* using Quicolor media, and they reported that these media provided reliable results within 4-9 hours.

Reporting the susceptibility results one day earlier can provide the beginning of the appropriate treatment for the patients, so it will contribute to reducing the side effects, to preventing from probable harmful impacts, to decrease mortality rate, to shorten the time of hospital stay, and to slow down the spreading of antimicrobial resistance. These positive effects will cause reduction of hospital costs, and will cause the low cost difference between the chromogenic and standard methods to turn into profit.^14^-^16^

In conclusion, our data showed that chromogenic Quicolor media provided reliable susceptibility results in enteric bacteria and staphylococci. However, the results were not in a convincing level of reliability in norfermentative group. We consider that further studies including higher number of nonfermentative bacteria are required to determine whether the chromogenic media are appropriate for this group of bacteria.
